# Endovascular treatment of symptomatic hepatic venous outflow obstruction post major liver resection

**DOI:** 10.1186/s12876-023-02876-3

**Published:** 2023-07-17

**Authors:** Patrick Ghibes, Christoph Artzner, Sasan Partovi, Florian Hagen, Silvio Nadalin, Gerd Grözinger

**Affiliations:** 1 Department for Diagnostic and Interventional Radiology, University Hospital Tuebingen, Hoppe-Seyler-Strasse 3, Tuebingen, 72076 Germany; 2 Department for Radiology, Diakonieklinikum Stuttgart, Stuttgart, Germany; 3 Interventional Radiology Section, Imaging Institute, Cleveland Clinic Main Campus, Cleveland, OH USA; 4 Department of General, Visceral, and Transplant Surgery, University Hospital Tübingen, Tübingen, Germany

**Keywords:** Hepatic venous outflow obstruction, Liver resection, Interventional treatment, Hepatic vein stenosis.

## Abstract

**Purpose:**

To evaluate efficacy, safety, and outcomes of endovascular treatment of hepatic vein stenosis post major liver resection.

**Methods:**

A retrospective data analysis was performed including all interventional treatments of hepatic vein stenosis post major liver resection since 2010. Post procedural course and clinical parameters including amount of ascites accumulation and relevant laboratory values were assessed during the follow-up period. Primary and primary assisted hepatic venous patency time were calculated.

**Results:**

Twelve patients (median age 55.5, IQR 49.75 to 61.5 years) undergoing a total of 16 interventions were included. Interventions were primary stent placement (n = 3), primary balloon angioplasty (n = 8), three re-interventions and two aborted interventions (no significant pressure gradient). Technical success was 100% (16/16). Permanent reduction and / or complete resolution of ascites was achieved in 72% (8/11). Laboratory parameters related to liver function did not show significant improvement after intervention. Median follow-up period was 6 months (IQR: 1.5 to 18 months). The median primary patency time for patients with balloon angioplasty was 11 months (IQR: 1.375 to 22.25 months) and assisted patency time was 13.25 months (IQR: 4.5 to 22.25 months). The median primary patency time for patients with angioplasty and stent placement was 1 months (IQR: 1.0 to 1.5 months) and assisted patency time was 2.0 months (IQR: 1.5 to 2.5months).

**Conclusion:**

An endovascular approach for the treatment of hepatic venous stenosis post major liver resection is safe and efficient to reduce and / or resolve refractory ascites. However, liver function parameters seem not to be improved by the procedure. Stent placement can be a reasonable option in patients with significant residual stenotic disease post angioplasty.

## Introduction

Hepatectomy is a commonly performed surgery with potential for cure in selected patients with malignant liver disease. Regarding the surgical procedure, a distinction can be made here between hemihepatectomy (resection of up to 4 liver segments) and extended hemihepatectomy (major liver resection) with resection of more than 4 liver segments and additionally the middle hepatic vein in some cases. Despite advances in hepatobiliary surgery, hemihepatectomy remains a procedure with major morbidity. Studies have shown mortality rates post hemihepatectomy of up to 5.8% and after major liver resection of up to 10.4% in Germany [1, 2]. A serious complication after hemihepatectomy is the so-called posthepatectomy liver failure (PHLF) [3–5]. Clinical symptoms include prolonged liver dysfunction with hyperbilirubinemia, intractable ascites, hepato-renal syndrome or sepsis [6, 7]. The etiology of PHLF is multifactorial. The main cause is a too small volume of the future liver remnant (FLR) after hepatectomy. Poor liver quality as well as impaired liver function already preoperatively and patient’s comorbidities may favor the development of liver failure. In recent years, investigations have shown that not only the remaining liver volume or liver quality is crucial for the regeneration of the liver parenchyma, but also inadequate portal-venous inflow or inadequate hepatic venous outflow can lead to liver failure with subsequent development of PHLF [4, 8]. The PHLF can be caused by excessive blood flow into the portal vein, which cannot be adequately drained by the remaining liver parenchyma via the hepatic venous system. Alternatively, PHLF may be caused by limited hepatic venous outflow due to stenosis of the remaining draining hepatic veins [9]. Both pathomechanisms lead to increased portal venous pressure with consecutive mechanical damage to hepatocytes leading to liver failure [10, 11].

The cause of a venous outflow problem after major liver resection is complex. In most cases the reduced liver volume leads to a rotation of the liver into the subphrenic space which may have a mass effect on the draining hepatic veins [12]. Only a few studies to date have examined the role of hepatic veins in the development of PHLF after major liver resection [12–14]. Up to now, there are no guidelines or study recommendations for the treatment of hepatic venous stenosis after major liver resection. Treatment of hepatic vein stenosis has been described particularly in the context after liver transplantation, where stenosis may occur in the area of vascular anastomoses. Interventional therapy is an attractive option for treating hepatic venous stenoses after transplantation [15]. Endovascular therapy options include balloon angioplasty or angioplasty with stent placement as reported in a limited number of cases after major liver resection [13, 16].

The aim of this study is a systematic retrospective analysis of interventional and clinical data describing procedural aspects, the efficacy as well as the outcome of endovascular treatment for hepatic venous stenosis post major liver resection. The technical details and complications of the procedure are analyzed including primary patency and primary assisted patency.

## Materials and methods

### Patients

Between 2010 and 2022, all patients who underwent endovascular treatment for symptomatic hepatic venous outflow obstruction post major liver resection were identified for this HIPAA-compliant, retrospective, institutional review board-approved study. The type of hepatectomy was classified according to the Brisbane 2000 Classification of Hepatic Resection as well as the so-called new comprehensive notation for hepatectomy (the “New world” terminology).

### Diagnosis of hepatic venous stenosis

**Fig. 1:**
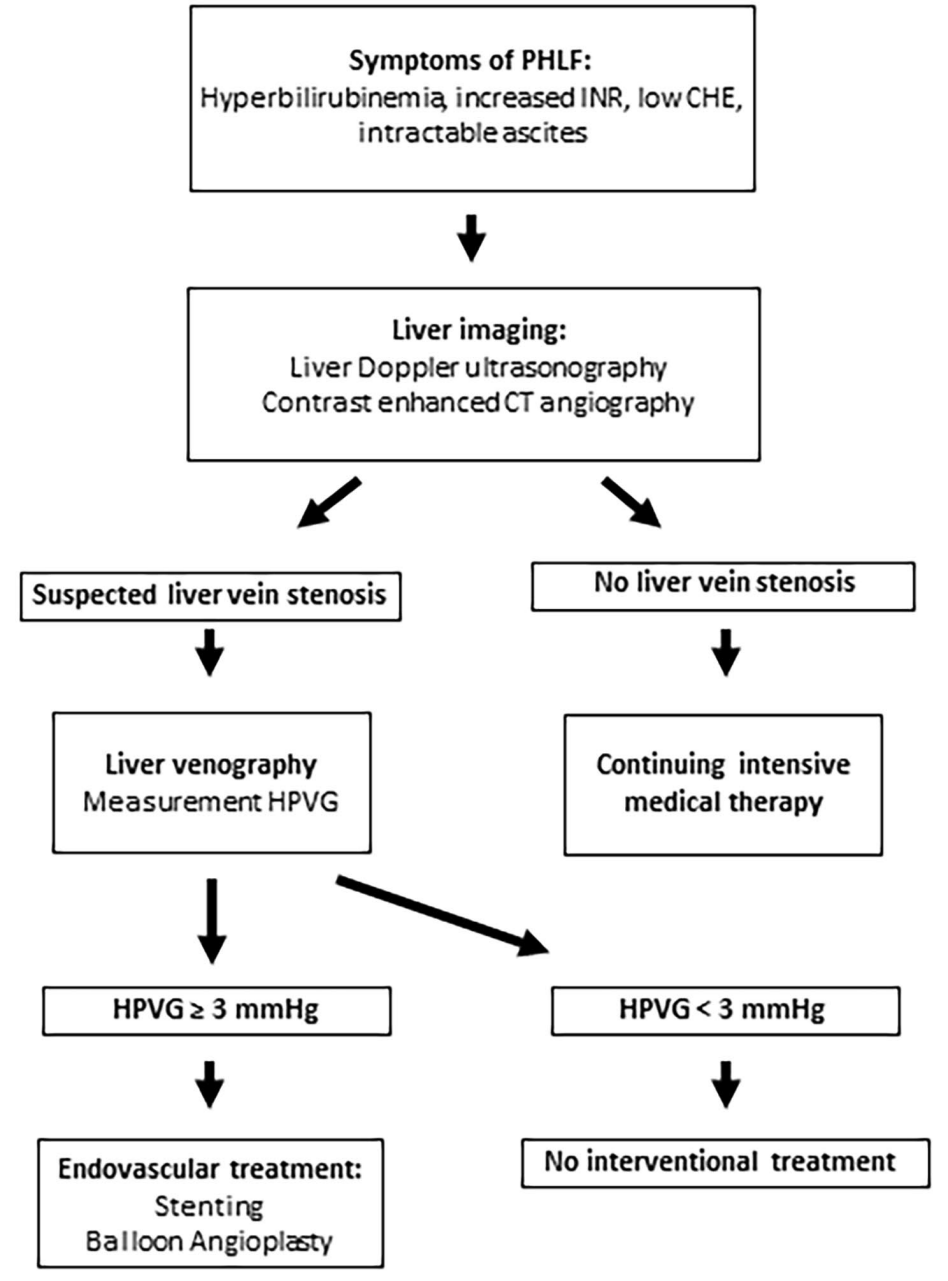
Flowchart for diagnosis and therapy of hepatic vein stenosis after major liver resection

**Fig. 2:**
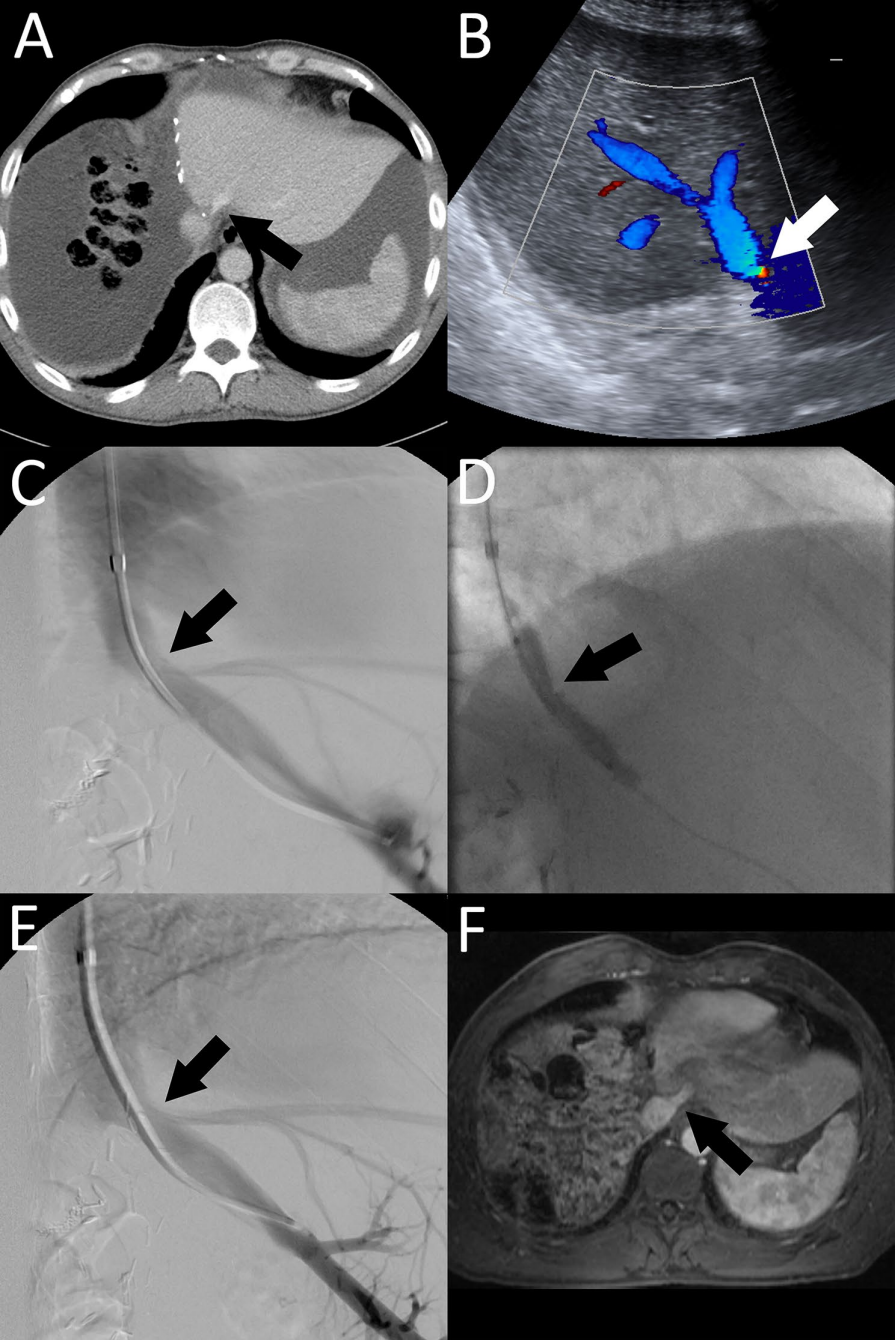
46-year-old male (patient no. 2) presenting with hepatic venous stenosis after extended right hepatectomy for cholangiocarcinoma. **A**: Axial postsurgical CT scan suggesting severe hepatic venous stenosis (arrow). **B**: Ultrasound exam confirms significant flow acceleration in the area of the suspected hepatic vein stenosis (arrow). **C**: Stenosis was found during venography and treated by balloon angioplasty using a 10 mm compliant balloon (**D**). **E**: Post-interventional result after balloon angioplasty without evidence of residual stenosis. **F**: MRI scan four months post intervention demonstrates a patent hepatic vein

**Fig. 3:**
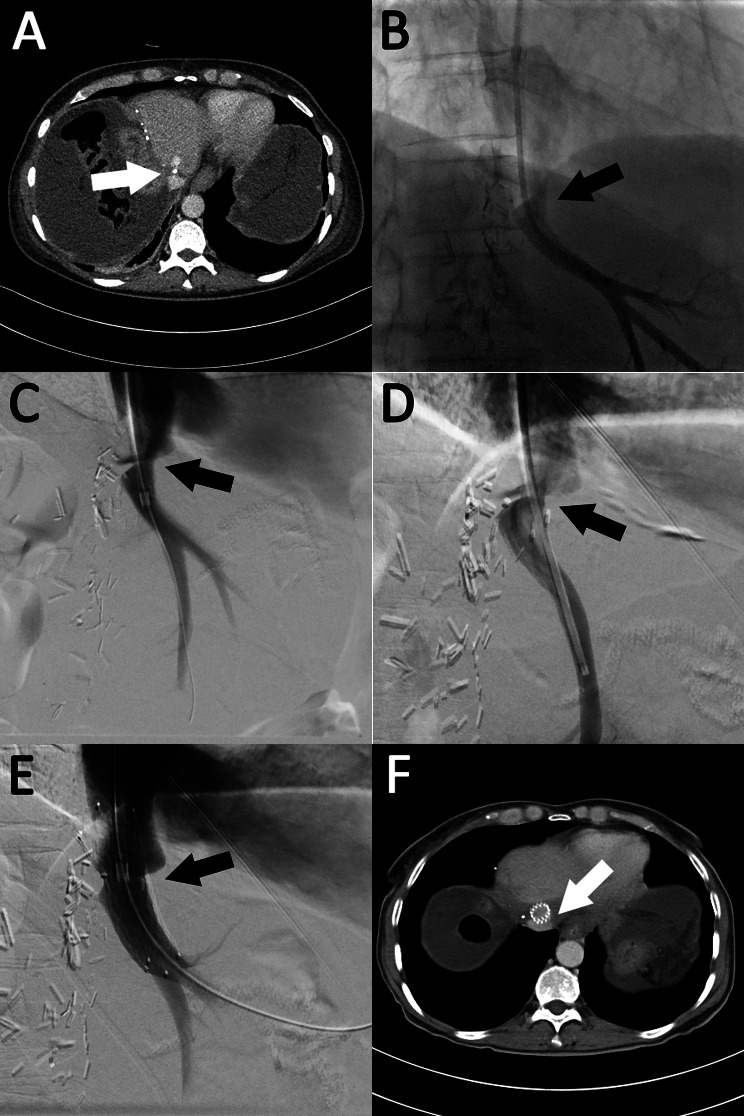
58-year-old male (patient no. 5) presenting with hepatic venous stenosis after extended right hepatectomy for cholangiocarcinoma. **A**: Axial presurgical CT scan suggesting severe stenotic disease (arrow) at the origin of the left hepatic vein. **B** and **C**: Stenosis was found venographically and treated by balloon angioplasty using a 10 mm compliant balloon. **D**: The stenosis remained present 13 days post first intervention with worsening ascites and therefore it was opted to pursue stent placement utilizing a self-expandable 14 × 40 mm nitinol stent (Sinus repo, Optimed, Ettlingen, Germany) (**E**). **F**: CT scan six months post second intervention shows a widely patent stent

The diagnosis of hepatic venous stenosis was based on clinical symptomatology of liver dysfunction and laboratory tests post major liver resection. Characteristic findings in patients with hepatic venous stenosis were refractory ascites and persistently poor liver laboratory parameters (INR, bilirubin, GGT, CHE, AST and ALT). An overview of the diagnostics is shown in the flow chart (see Fig. 1). In these cases, further imaging with liver ultrasound with Doppler and contrast enhanced CT angiography were indicated to assess for vascular complications (portal vein occlusion, stenosis of the hepatic artery or hepatic vein). The definitive diagnosis of hepatic venous stenosis by imaging criteria can be challenging and therefore it is typically suspected after exclusion of other vascular causes. In case of visible luminal narrowing of the hepatic vein (see Figs. 2 and 3) or vastly abnormal waveform on power Doppler as well as high-grade clinical suspicion, the indication for diagnostic venography with the possibility of intervention was made after interdisciplinary discussion between liver surgeon, interventional radiologist and intensivist. During venography, it was possible to measure the pressure gradient between intrahepatic vein and Vena cava inferior (HPVG) to confirm the diagnosis of hepatic vein stenosis if the pressure gradient along a suspected stenotic area was increased (> 3–5 mmHG) [17–19].

### Procedural technique

The actual current standard procedure is described below (see also Fig. 1).

In all patients, access to the hepatic vein was established by ultrasound guided percutaneous access of the right internal jugular vein (n = 11) or femoral vein (n = 2). A 6 or 7 French sheath was placed and the hepatic vein was selected using a combination of an angled catheter (usually multipurpose configuration, such as MPA catheter) along with a guidewire (usually Bentson or Glidewire) combined with careful injection of contrast medium under fluoroscopy guidance. Venogram of the hepatic vein and inferior vena cava was obtained from different angles to localize the stenosis and to determine the reference vessel diameter. Pressure measurements were performed in the hepatic vein as well as in the inferior vena cava in order to establish the hemodynamic relevance of the visualized stenosis by means of pressure gradients. Pressure gradients greater than 3 mmHg were considered to require therapy. If the pressure gradient was borderline (between 2 and 3 mmHg) the entire clinical picture including venographic findings were taken into consideration to decide whether to proceed with endovascular intervention. In case of a confirmed stenosis, balloon angioplasty was performed as a first step using a compliant balloon with a diameter equal to the pre-stenotic hepatic vein. Inflation time were up to one minute and could be repeated as necessary to minimize the risk of iatrogenic thrombosis in the hepatic vein. In cases of residual stenosis post venoplasty, an angioplasty balloon with a 1-mm larger diameter than the stenosis diameter was used to dilate the stenotic area. Stent placement was reserved for patients with significant residual stenosis post angioplasty or patients who had already undergone balloon angioplasty presenting with recurrent hemodynamically relevant hepatic venous stenotic disease. Stent placement was performed using a balloon-expandable bare-metal stent that had a closed-cell design (e.g. Palmaz Blue, Cordis, Dublin, Ireland) (n = 2) or a self-expandable nitinol stent (e.g. Sinus repo, Optimed, Ettlingen, Germany) (n = 6). The stent size slightly exceeded the target diameter of the vessel to ensure appropriate fixation and molding to the venous wall. In the next step, a compliant balloon with a diameter suitable for the vessel was inflated inside the stent in order to achieve the desired final inner stent diameter. Final venogram was performed to confirm brisk contrast flow through the reconstructed hepatic vein. Ultrasonography of the abdomen was subsequently performed to exclude intra-abdominal bleeding. Post procedure patients with low bleeding risk received unfractioned heparin for 72 h with a target partial thromboplastin time of 50 to 70 s followed by weight-based low-molecular-weight heparin for 3 weeks.

### Follow-up

Post procedure liver ultrasound with Doppler was performed on day 1 to determine liver perfusion and quantify the amount of ascites. Laboratory data related to liver function (PT/INR, bilirubin, and hepatic function panel including liver enzymes) were monitored daily during the stay in the intensive care unit. Patients with increasing ascites or worsening liver function received CT angiography to evaluate for possible hepatic venous restenosis and to exclude other complications.

After discharge patients had regular follow-up in person. Outpatient visits every three months for abdominal ultrasound. If the amount of ascites increased, patients were immediately admitted from clinic.

### Definition and analysis

Technical success was defined as successful completion of the procedure without evidence of a pressure gradient between the hepatic vein and inferior vena cava and without venographically visible hemodynamically relevant stenosis upon completion of the procedure. Clinical success was defined as an improvement in symptoms related to sequela of portal hypertension (specifically refractory ascites) as well as improvement of laboratory values related to liver function.

Primary patency was defined as the post interventional procedure time interval without signs of recurrent hepatic venous stenosis. Assisted primary patency was defined as the symptom-free time after the initial endovascular hepatic venous intervention including subsequent interventions in case of re-stenosis.

We categorized serious adverse events based on the Cirse (Cardiovascular and Interventional Radiological Society of Europe) quality assurance document and standards for classification of complications [20].

## Results

Between 2010 and 2022, 12 patients underwent 16 hepatic venous interventions after major liver resection (see Table 1). The median age of the patient group was 55.5 years (IQR 49.75 to 61.5 years). The indication for hemihepatectomy was primary liver malignancy (cholangiocarcinoma (CCA) n = 7, liver sarcoma n = 1), 3 patients had metastases from a non-hepatic primary malignancy (colorectal liver metastasis (CRLM) n = 2, melanoma n = 1) and one patient underwent hemihepatectomy secondary to liver parenchymal necrosis in the setting of abdominal trauma. All patients underwent right extended hemihepatectomy (major liver resection). In 7 of 12 patients, the middle hepatic vein was also resected. Eight patients underwent primary balloon angioplasty and three patients underwent primary stent placement. In one patient, interventional therapy was not performed secondary to lack of a significant pressure gradient. Four patients had suspected recurrence of hepatic venous outflow obstruction: Two patients underwent stent placement after primary balloon angioplasty, one patient underwent stent extension after primary stent placement. One patient showed no significant pressure gradient in the case of suspected restenosis and therefore no endovascular treatment was performed. Demographic and procedural details are shown in Tables 1 and 2. In most cases, hepatic vein stenosis was suspected during the inpatient stay post hemihepatectomy. The median time between surgery and first intervention was 31.5 days (IQR 26 to 45 days). Only one patient showed refractory ascites at an outpatient follow-up appointment 2 months after hemihepatectomy with otherwise unremarkable liver function parameters.

**Table 1:** Baseline characteristics

Patient number	Age [year]	Sex	Primary disease	Type of Hepatectomy	First intervention	Second intervention
1	61	m	cholangiocarcinoma (CCA)	NWT: H45678 Brisbane: Right Trisectionectomy	balloon angioplasty	stent placement
2	46	m	CCA	NWT: H145678 BRISBANE: right Trisectionectomy	balloon angioplasty	
3	63	m	Liver sarcoma	NWT: H145678 BRISBANE: right Trisectionectomy	balloon angioplasty	
4	42	m	Liver metastasis uveal melanoma	NWT: H145678-MHV BRISBANE: right Trisectionectomy	stent placement	
5	58	w	CCA	NWT: H145678-MHV BRISBANE: right Trisectionectomy	balloon angioplasty	stent placement
6	57	w	CCA	NWT: H145678-MHV BRISBANE: right Trisectionectomy	stent placement	stent placement
7	51	w	CCA	NWT: H145678-MHV BRISBANE: right Trisectionectomy	balloon angioplasty	
8	53	m	CCA	NWT: H145678 BRISBANE: right Trisectionectomy	balloon angioplasty	
9	79	m	CCA	NWT: H145678-MHV BRISBANE: right Trisectionectomy	balloon angioplasty	
10	54	w	colorectal liver metastases (CRLM)	Right (IV-VII) NWT: H45678-MHV Brisbane: Right Trisectionectomy	balloon angioplasty	
11	67	m	CRLM	NWT: H145678-MHV BRISBANE: right Trisectionectomy	Venography	
12	16	w	Liver trauma	Right (IV-VII) NWT: H45678 Brisbane: Right Trisectionectomy	stent placement	Venography

**Table 2:** Procedural details

Patient number	Number of interventions	Access	Technical success	Complications	Type of balloon	Type of stent
1	1	right VJI	1	0	Passeo 7/ 40 mm Passeo 8/ 40 mm Armada 9/ 40 mm	
1	2	right VJI	1	0	Armada 14/ 40 mm	Sinus pro 14/ 40 mm
2	1	right VJI	1	0	Rival 10/ 40 mm	
3	1	right VJI	1	0	Passeo 6/ 40 mm Passeo 7/ 40 mm	
4	1	right VJI	1	0	Fox Cross 10/ 40 mm	Protégé GPS 12/ 40 mm
5	1	right VJI	1	0	Passeo 10/ 40 mm	
5	2	right VJI	1	0	Passeo 10/ 40 mm	Sinus Repo 14/ 40 mm
6	1	right VJI	1	0	Armada 3/ 40 mm	Palmaz Blue 5/ 12 mm Sinus Repo 6/ 15 mm
6	2	right VJI	1	0	Passeo 10/ 20 mm Viatrac 5/ 40 mm	Dynamic Renal 6/ 12 mm Sinus Repo 14/ 40 mm
7	1	right VJI	1	0	Armada 10/ 60 mm Armada 14/ 40 mm	
8	1	right VJI	1	0	Passeo 7/ 40 mm	
9	1	right VJI	1	0	Passeo 8/ 40 mm Passeo 10/ 60 mm	
10	1	right VJI	1	0	Passeo 9/ 40 mm	
11	1	right VJI	-	0	-	-
12	1	right VF	1	0	Passeo 10/40 mm	Sinus Repo 14/40 mm
12	2	left VF	-	0	-	-

### Technical success

Technical success was achieved in all interventions (100%) who were found to have hepatic venous stenosis based on intra-procedural measurements of pressure gradients. Two Interventions were considered as technically successful, even though no endovascular treatment was necessary since there was a lack of a significant pressure gradient in these two cases (< 5mmHg). In all procedures, hepatic venous access was feasible via the percutaneous access of the right internal jugular vein (n = 14) or common femoral vein (left n = 1, right n = 1).

The pressure gradient between the hepatic vein and inferior vena cava was determined in 12 of the 16 interventions (see Table 3). In 12/12 patients, a reduction of the pressure gradient could be accomplished, however in three patients the post-interventional pressure gradient remained elevated (> 5 mmHG).

**Table 3:** Measured pressure gradients between hepatic vein and inferior vena cava pre- and post-intervention. Of note, in two patients no significant pressure gradients were appreciated and therefore no endovascular interventions were performed

Patient	Intervention	Pressure gradient pre-interventional [mmHg]	Pressure gradient post-interventional [mmHg]
1	1	25	9
4	1	20	10
5	1	2	1
5	2	4	1
6	1	20	3
7	1	19	13
8	1	6	4
9	1	7	3
10	1	7	3
11	1	no significant pressure gradient (4 mmHg)	-
12	1	7	1
12	2	no pressure gradient (0 mm Hg)	-

### Clinical success

A sustained decrease in ascites was achieved in 8 of 11 patients (73%). In two patients, a second intervention was necessary to achieve a reduction in the amount of ascites. Two further patients did not show long term decrease in ascites production. One patient developed an abscess at the resection site after hemihepatectomy and consecutive significant ascites production with indication for drain placement.

Laboratory parameters in a time period of one week before and one week after the intervention were collected with the goal to detect improvement associated with the endovascular treatment of hepatic venous stenotic disease (Table 4). The INR showed a non-significant increase post intervention (1.53 ± 0.70 to 1.58 ± 0.78, p-Value 0.526). Bilirubin (6.41 ± 7.02 to 5.86 ± 7.10, p-Value 0.753), GGT (94.58 ± 61.98 to 78.00 ± 38.46, p-Value 0.224) and ALT (34.00 ± 24.20 to 37.62 ± 43.41, p-Value 0.611) did not reveal any significant post intervention improvement, either. Cholinesterase (CHE) was not determined in all patients as standard. CHE was determined pre-interventionally in only 4 patients and was decreased in all cases. Post-interventionally, CHE was determined again in only one patient. In this case, there was no significant increase of CHE after intervention.

**Table 4:** Laboratory parameters to assess liver function pre- and post-intervention. Arithmetic mean plus/minus standard deviation and p-values are listed

	Pre-interventional	Post-interventional	p-value
INR	1.53 ± 0.70	1.58 ± 0.78	0.526
Bilirubin [mg/dl]	6.41 ± 7.02	5.86 ± 7.10	0.753
GGT [U/I]	94.58 ± 61.98	78.00 ± 38.46	0.224
ALT [U/I]	34.00 ± 24.20	37.62 ± 43.41	0.611

### Patency time

**Fig. 4:**
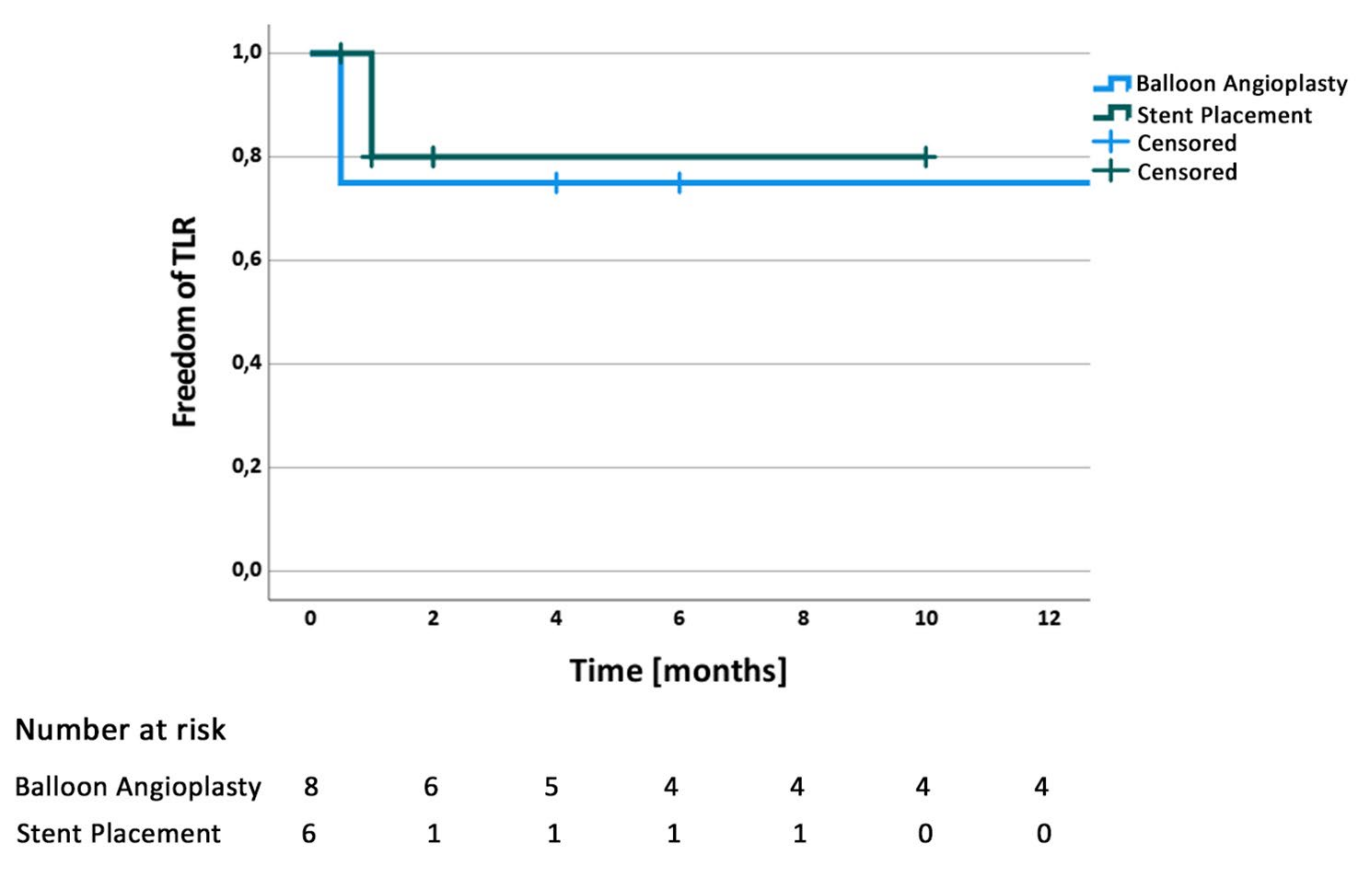
Kaplan-Meier curve demonstrating freedom of target lesion revascularization (TLR) post balloon angioplasty and post stent placement

The median clinical follow-up period was 6 months (IQR: 1.5 to 18 months). Two patients dropped out of the follow-up program without recurrence of hepatic outflow obstruction or recurrence of their underlying malignancy. Nine patients died in the short-term post intervention due to progression of their underlying malignancy (see Fig. 4). One patient underwent liver transplantation for liver parenchymal necrosis after abdominal trauma. The two patients without significant pressure gradient were excluded from the study calculations since no endovascular interventions were performed in these patients. For patients with balloon angioplasty the primary patency time was 11 months (IQR: 1.375 to 22.25 months) and assisted patency time was 13.25 months (IQR: 4.5 to 22.25 months). Patients with stent placement had a primary patency time of 1 months (IQR: 1.0 to 1.5 months) and an assisted patency time of 2.0 months (IQR: 1.5 to 2.5months).

### Complications

There were no minor or major complications related to the endovascular procedure appreciated.

## Discussion

This study investigated the experience, safety and outcomes of endovascular treatment in PHLF after major liver resection due to symptomatic venous outflow obstruction. Due to the advances in hepatobiliary surgery, there is an increasing number of performed hemihepatectomies and the procedure is associated with a known risk of postoperative liver failure [21]. The risk of PHLF has been citated as high as 32% in some studies with a mortality risk of up to 40% [5, 22].

The small-for-size syndrome (SFSS) is often used to describe liver failure in the setting of transplant [11, 13, 16, 23]. The reduced liver volume leads to a mismatch between liver remnant and metabolic demand with consecutive symptoms of liver failure. PHLF after major liver resection may also be due to an insufficient liver volume, the symptoms are similar to the small-for-size syndrome after liver transplantation. In rare cases, stenosis of the hepatic veins also occurs after major liver resection, which may become symptomatic in cases of higher-grade stenosis [12, 7, 22]. As a result, portal venous pressures increases leading to parenchymal stasis with mechanical damage to hepatocytes and subsequent organ failure [10, 16, 23, 24]. There are a variety of factors causing hepatic venous outflow challenges in the post major liver resection patient population. Migration of the liver into the subphrenic space with consequent obstruction of the hepatic veins has been reported [12, 13, 25, 26]. Alternatively, postsurgical adhesions or strictures may lead to constriction of the hepatic veins [13]. Liver-related constriction of the hepatic veins due to hypertrophy and consecutive rotation/ twisting in the course of postsurgical regeneration may also cause delayed hepatic venous stenosis or may contribute to an already existing outflow problem [9, 24, 27]. This phenomena fits well with the observations in this study as a focal constriction was often seen at the junction between the hepatic veins and the inferior vena cava. Evaluation of the study population also showed that the median hepatic vein was resected in the majority of patients. This may represent an additional risk factor, as in this case only one hepatic vein remains. Due to the close anatomical relationship between the middle and left hepatic veins, surgical removal of the middle hepatic vein may additionally lead to iatrogenic constriction of the left hepatic vein. This can lead to the development of PHLF, especially in the absence of collateral or additional draining hepatic veins [28, 29].

The number of studies assessing hepatic venous stenosis post hemihepatectomy are limited and to the best of our knowledge a few case reports exist [13, 26, 30]. In contrast, several larger studies have evaluated endovascular therapy of hepatic venous stenosis post liver transplantation. The cause in the transplant population likely differs compared to the post hemihepatectomy population since scarring at the anastomotic site is typically the cause for post liver transplant hepatic venous stenotic disease.

Most patients with suspected hepatic vein stenosis showed postsurgical liver failure and ascites which did not improve with best medical management. The suspicion of a hepatic vein stenosis prior to endovascular therapy in this study was based on the exclusion of other possible causes through imaging and laboratory values since the definitive diagnosis of hepatic venous stenosis can be challenging with non-invasive imaging [17, 31]. Ultrasound visualization of the hepatic veins can be limited particularly in the immediate postsurgical setting and CT may enable suggestion of hepatic venous stenotic disease without quantitative flow measurement across the stenotic area [11, 32].

A total of 16 interventions were performed in this study with a technical success rate of 100% which is similar to similar studies in the liver transplant patient population [15, 33]. The pressure gradient was determined in 12 of 16 interventions. In 75% of the cases there was a significant decrease in the pressure gradient post endovascular intervention. In three patients the post-interventional pressure gradient was still elevated (above 5 mmHg) [34].

Initial clinical success with decrease in the amount of ascites was achieved in 8/11 patients undergoing technically successful endovascular interventions. Two patients passed away shortly after intervention due to complications not related to the interventional procedures. Evaluation of laboratory parameters showed no significant improvement post intervention. However, a significant change in laboratory parameters in the liver disease population is not necessarily expected. Patients with liver cirrhosis and portal hypertension undergoing a transjugular intrahepatic portosystemic shunt (TIPS) creation may show a decrease in ascites without a change in laboratory parameters [35]. Liver parenchymal damage has probably already occurred and cannot be reversed in the short term with optimization of the liver vasculature including hepatic venous outflow improvement [11, 23, 26]. Therefore patients post hemihepatectomy who have refractory ascites and adequate liver function may benefit from endovascular treatment of the hepatic venous system. For example, patient 3 with adequately recovered liver function had continuous ascites at an outpatient follow-up appointment 2 months post hemihepatectomy. With suspected hepatic vein stenosis and successful endovascular balloon angioplasty, the patient was subsequently symptom-free with regression of ascites. Overall primary reduction of ascites was achieved in 8 of 11 of patients receiving endovascular treatment which equals 73%.

In this patient population the outcomes were poor due to the underlying malignancy. Recurrence or newly diagnosed metastatic disease occurred several months after the intervention and many of these patients were unable to receive further anti-neoplastic therapy due to their general clinical with a subsequent dismal prognosis.

For the choice of interventional therapy procedure, the question arises whether primarily balloon angioplasty or stent placement should be performed. In this patient population, the majority of primary interventions were balloon angioplasty. Studies in patients with hepatic vein stenosis after liver transplantation revealed that primary stent placement (with balloon angioplasty) has higher patency time compared to balloon angioplasty alone [36–39]. Our datasets show a significantly longer patency time after primary balloon angioplasty. However, this difference in patency time may be explained by the smaller study population, the overall worse prognosis due to underlying malignant disease and a lack of experience with stenting of venous vessels in previous earlier studies. In the critically ill hemihepatectomy patient population re-stenosis should be avoided and therefore primary stent may be the favorable approach in case of insufficient result after balloon angioplasty. Based on our experience as primary stent a self-expanding Nitinol stent is a reasonable option because of the conical geometry of the hepatic vein and the pronounced respiratory mobility requiring a more flexible stent design which enables adaption to complex geometry. Due to the respiratory mobility there is a risk of stent migration which can be reduced by using a flexibly adaptable stent design, such as the self-expanding Nitinol stent.

The study has several limitations including the retrospective study design. A prospective study design was not possible because of the small number of patients (12 patients in 12 years) and the associated long recruitment period. Second, the number of patients is small and therefore statements about the preferred interventional technique should be made with caution (primary balloon angioplasty versus primary stent placement). Most patients were referred to the quaternary medical center from outside hospitals. Therefore, primary and assisted primary patency time was calculated based on the time period between the first procedure and the last available follow-up. These patency time may potentially differ from the actual patency time due to the considerable loss of follow-up examinations.

## Conclusion

Endovascular treatment of symptomatic hepatic venous stenosis post hemihepatectomy is safe and efficient. Balloon angioplasty or endovascular stents are the methods of choice. Intervention is indicated in case of HVPG of more than 3 mmHG. To avoid short-term re-intervention Stent placement can be a reasonable option in patients with significant residual stenotic disease post angioplasty.

## Data Availability

The datasets used and analyzed during the current study are available from the corresponding author on reasonable request.

## List of abbreviations

CCA: Cholangiocarcinoma
Cirse: Cardiovascular and Interventional Radiological Society of Europe
CRLM: Colorectal liver metastases
PHLF: Post hepatectomy liver failure
SFSS: Small-for-size syndrome
TIPS: Transjugular intrahepatic portosystemic shunt
